# Nursing students’ perception of gender-defined roles in nursing: a qualitative descriptive study

**DOI:** 10.1186/s12912-022-00876-4

**Published:** 2022-05-04

**Authors:** Mirko Prosen

**Affiliations:** Department of Nursing, Faculty of Health Sciences, University of Primorska, Polje 42, 6310 Izola, Slovenia

**Keywords:** Sexism, Stereotypes, Male nurses, Education, Equality, Drawing method

## Abstract

**Background:**

Gender stereotypes influence both women and men who work as nurses as well as individuals considering nursing as a profession. The aim of this study was to explore male and female nursing students’ experiences and perspectives regarding gender-defined roles as well as how they view their future professional roles.

**Methods:**

The study used a descriptive qualitative design. A convenience sample included 72 full-time second- and third-year undergraduate nursing students. Data were collected in 2017 using a self-administered qualitative questionnaire consisting of 11 essay-type questions. As an additional data collection method, a drawing method was applied. The data were analysed by content analysis separately for the male and female perspectives.

**Results:**

Altogether, eight themes emerged, with half explaining the male and the other half the female students’ perspectives. Students choose nursing for altruistic, opportunistic and organisational reasons. Among the female students, another reason “family and social incentives” was also identified. Female students’ vision of their future professional role included altruism and positive representations of ‘being a nurse’, whereas the male students’ vision included management and leadership, and technical aspects of nursing.

**Conclusions:**

Both male and female nursing students face the limits of their own gender roles, yet they are prepared to challenge these gender-based perceptions. Tackling stereotypes and raising public awareness, using gender-inclusive language and strategies for recruitment, ensuring gender diversity in nursing teams and revising the nursing curriculum where appropriate, are just some of the implications that arise to help overcome gender differences.

## Background

Despite the huge challenges the nursing profession has faced since the start of 2020, which coincided with the International Year of the Nurse and the Midwife, the year 2020 was also a time to reflect on the profession and its future development. One issue which initially seems out of place in discourses in the twenty-first century, yet is very important in terms of professionalisation, is gender-defined roles (at least how they are perceived) in nursing. It is a fact that throughout history nursing has been a female-oriented profession. Even Florence Nightingale envisioned nursing as a profession most suitable for women [1–4]. She saw it as an extension of mothering and therefore felt females were more appropriate for caring. Men in nursing were isolated from the profession, psychiatry being the one field in which men were desirable [3, 4]. However, Nightingale’s vision and work must be understood in the societal context of her era when the Victorian view on gender-specific roles prevailed.

Around the world, men quite rarely decide on a career as a nurse. In Western countries, male nurses rarely exceed 10% [5, 6]. The proportion varies between 1 and 2% in China [4, 7] to 16.7% in Spain [7]. Although some progress towards greater gender diversity has been made, males in nursing continue to be underrepresented [8]. In Slovenia, the proportion between female and male nurses with a bachelor’s degree has changed over the years in favour of male nurses, while it has remained stable among Registered Nursing Assistants (RNA). Between 2010 and 2019, the average share of female nurses holding a bachelor’s degree ranged from 93.83% in 2010 to 88.66% in 2019 and 6.17 to 11.34% for male nurses, respectively. The average share of male nurses working as an RNA between 2010 and 2019 was 13.77% [9]. The large increase in male nurses in bachelor’s degree programmes over the last decade in Slovenia can be attributed to several factors, but especially the establishment of new bachelor nursing programmes, changes in the public image of nursing, and the greater employment possibilities and hence the job security that nursing brings. These are also reasons often identified as explaining why male nursing students, besides the desire to care for and help people in need, decide on nursing [10].

Gender and gender-defined roles are socially constructed and change over time. Gender refers to the role and responsibilities as well as the opportunities that stem from the biological fact of being male or female. This includes beliefs, values, attitudes, representations, prejudices, stereotypes, social norms, obligations and prohibitions about men’s and women’s behaviours, sexualities, relationships and thereby affects professional life [7]. The myth surrounding female nurses and femininity and male nurses and masculinity is misleading, particularly when there is no strong evidence that gender affects caring behaviour [11]. However, the notion of femininity is often challenged by gender stereotypes and thus stigmatised and discriminated [4, 8, 10]. The perception that nursing comes ‘naturally’ to women due to their biological and reproductive role is seen as ‘unnatural’ for the men who engage in a ‘feminine-oriented’ profession [5, 7]. This idea further generates stereotypes associated with gender roles linked to women and men who enter the nursing workforce and influences individuals (male or female) who are considering nursing as a profession. This gender-defined image before enrolling in a nursing programme means that many male students experience prejudice in terms of a lack of support from the social environment for selecting nursing as their career choice, with male peers questioning their masculinity and heteronormativity, and being discouraged by the inability to join in a male tradition in nursing or to find a male nurse role model with whom to identify. Ross [3] argues that men in nursing continue to be discriminated against due to the above-mentioned socially constructed gender roles and norms, which initially prevented and now discourage men from entering the nursing profession. Similar can be found after enrolment when male students’ answers are overlooked in favour of female students’ answers while discussing women’s health topics, feminisation of the nursing curriculum and the underrepresentation of males in nursing study literature [6, 7]. Despite these challenges, during their student years and later in the nursing profession males entering this ‘female-oriented’ profession develop strategies (e.g. specialised/technical wards) to help them retain their ‘masculinity’ [3, 7, 8]. However, female nurses must also deal with their own challenges related with gender roles within a health system entailing patriarchy and male dominance/female subordinated relationships that dictate their professional role and affect professionalisation of nursing [12].

The aim of the study was to explore male and female nursing students’ experiences and perspectives on gender-defined roles as well as how they view their future professional role.

## Methods

### Study design

A descriptive qualitative study was used since such studies are based on the general premises of constructivist inquiry and assume a naturalistic perspective. It’s a design particularly used in examining health care and nursing-related phenomena [13]. Qualitative description provides a direct description of the phenomena under study, staying close to the perspective and interpretation of the participants in a particular space and time. This research design is appropriate for research questions that focus on exploring the who, the what, and the where of events or experiences and gaining insights from informants regarding poorly understood phenomenon [13]. Therefore, the method seemed very suitable for helping to understand nursing students’ perception of gender-defined roles in the profession and for the data collection approach that was utilised. The Consolidated Criteria for Reporting Qualitative Research (COREQ) were used [14].

### Sample and setting

The research took place among undergraduate nursing students at the Faculty of Health Sciences, University of Primorska (FHS UP) in Spring 2017. The FHS UP is a publicly-funded higher education institution established in 2002. In Slovenia, a nursing degree is obtained after the completion of a 3-year undergraduate study programme (180 ECTS). This programme is based on various directives and regulations pursuant to EU sectoral directives, national legislation and initiatives of international professional associations. However, the entry point for nursing education in Slovenia can be high school (secondary, vocational education), where participants may attain the position of RNA [15].

A convenience sample was used in the qualitative study instead of traditional purposive sampling due to the data collection method used as well as the efficiency associated with convenience sampling [13]. Since this was not a quantitative study, representativeness was not the issue in the classical sense, as was the information power of the data describing the phenomenon, especially through the use of drawing method [16]. The convenience sample included 72 (46.75%) full-time second- and third-year students enrolled in the undergraduate Nursing programme in the 2016/2017 academic year (among 154 enrolled in that academic year). Second- and third-year nursing students were chosen due to their immersion in clinical settings.

### Data collection

The data were collected using a paper-and-pencil self-administered qualitative questionnaire [13] because this kind of data collection not only increases the sample size but also supports anonymity and the drawing method [17, 18] as an additional approach to collecting data. The use of qualitative questionnaires as a method of data collection is not new. The method was originally used as an ethnological research method to document and collect material about everyday life. Qualitative questionnaires have much in common with other qualitative methods, such as diary entries, because both consist of memories, personal perspectives and experiences, however qualitative questionnaires are much more guided [19].

The questionnaires were distributed after lectures and no time limit was given to complete the questionnaire. Two types of questionnaires were used, one addressing the female and the other the male nursing students. The differences in the questions in these two questionnaires were minor, i.e. addressing either the male or female perspective. The questionnaires consisted of a series of open-ended questions (Table 1) in which the last question was followed by half a blank page, asking the students to draw how they see themselves in nursing. They were later asked to explain their drawing.

**Table 1:** Open-ended questions

Open-ended questions	Included in the questionnaire for male/female nursing students
What was your reason for choosing nursing?	Male/Female
Did you have any second thoughts entering a profession in which females prevail? Was this a relevant factor?	Male
What’s your perspective on males in the nursing profession? What’s your experience of interacting with them?	Female
Did you notice any differences in the treatment of female and male nursing students during your clinical placement?”	Male/Female
Did you notice in clinical settings or during your education the existence of any stereotypes associated with males/females in nursing?	Male/Female
Did you feel the patients acted differently towards you due to your gender (either a positive or negative experience)?	Male/Female
Do you feel the nursing curriculum is focused more on women’s perspective than men’s?	Male/Female
Do you feel bothered that in the profession we talk about nurses (in the female grammatical form)?	Male/Female
Is this taken for granted today?”	Male/Female
Did you experience any barriers as a male/female nursing student within the class?	Male/Female
Did you experience any barriers as a male/female nursing student in the clinical practice or during your study at the faculty?	Male/Female
Please describe your own thoughts and experiences regarding the topic (optional).	Male/Female

The concepts of social reality are not easily interpreted, especially when exploring complex phenomena like gender issues in nursing. The drawing method, as a participatory research method, was chosen as it allows its creator to simplify the complexity of the observed socially constructed reality [20] and thereby to escape the linearity of the spoken or written word [21]. The draw-and-write technique is not a new method of data collection and has been used in a variety of settings as either a stand-alone method or as part of a wider set of research methods [17]. It is far from a simple method because it not only involves participants in creating drawings, but also writing about what their drawing means. The researcher-participant collaboration in this method helps with understanding the drawing’s meaning [22].

### Data analysis

All handwritten questionnaires completed by students were typed into a word processor. Each questionnaire was assigned a unique code (consisting of age, gender /F or M/, year of study, full /F/ or part-time /P/ student, and a sequential number to distinguish from similar ones). The data (written and visual) obtained from the study were analysed using the qualitative content analysis method with an inductive approach. As a research method, it is often referred to as a systematic and objective approach to describing and quantifying phenomena [23] and as a common analysis strategy in the qualitative descriptive method [13]. The analysis was conducted separately for the male and female students’ perspectives to ensure a clear and more detailed understanding of the phenomena. The data were analysed using the qualitative data analysis software NVivo ver. 12. The data analysis was performed in Slovenian. Quotes in the manuscript were translated into English and proof-read by a professional translator.

The content analysis process of the written text had three main phases: preparation, organisation and reporting. In phase one, we read and reread the questionnaires to make sense of the data. Phase two entailed open coding and grouping of the coded material based on shared concepts and a final abstraction. In the reporting phase, the findings were described following the content of the themes and addressed the research questions [13, 24].

Where participants drew a representation of themselves in nursing, these drawings were scanned in jpg. Format and assigned the same unique code as given to the interview and uploaded together with an accompanying text in a password-protected computer file. The drawings were analysed using the four-step content analysis method suggested by Rose [17]: (a) finding images relevant to the research question; (b) devising categories for coding, e.g. attaching a set of descriptive labels or ‘categories’ to the images; (c) coding the images; and (d) analysing the results.

### Rigour and trustworthiness

Since integrity in qualitative research must be ensured, we paid attention to its credibility, dependability, confirmability, transferability and authenticity [13]. To make the findings more transferable, the whole research process and findings are documented in detail. The questionnaires were pilot-tested to check the understanding and clarity of the questions, with the results revealing no changes were needed. Before initiating the study and after thorough consideration and elaboration with peer experts in methodology, it was decided that the researcher would be present in the room during the data collection to prevent groupthink and support an individual approach [24]. In no way students were coerced in participating in the study or any other way influenced. The role of the researcher in this context was clear.

The research steps were peer-reviewed prior to commencing the study. After the author performed content analysis, the findings were cross-checked. However, this process of peer-reviewing occurred early on in the analysis of the drawings since it is important that this is done already in the coding phase to ensure a high level of replicability [17]. During the analysis, we constantly reflected on the findings by continually comparing them with the literature. This constant reflection also made us aware of the existing limitations and possible biases arising from the subjective perspective.

### Ethical considerations

Before the study commenced the study was approved by the Commission for Scientific Research and Development of the FHS UP. The Commission assessed all the aspect of the study including ethical perspective. Participation in the study was anonymous and voluntary. All students were fully informed about the study’s aim and the methods used to gather data before the questionnaires were distributed. Written informed consent was obtained, and those who agreed to participate took and completed the questionnaire. Those not wishing to participate were free to leave the classroom. The students were told to try to answer all the questions, including the drawing, but there would be no consequences if they did not. Names and places mentioned by the students were anonymised in the questionnaires.

## Results

The sample of 72 participants comprised 58 (80.56%) female students and 14 (19.44%) male students. These included 33 (45.83%) second-year and 39 (54.17%) third-year undergraduate nursing students. With an average participant age of 22.34 years, 45 (62.50%) of them were residing in a rural and 27 (37.50%) in an urban area. Before enrolling in the undergraduate nursing programme, 55 (76.39%; 43 female and 11 male students) had graduated from a vocational school as an RNA while 17 (23.61%; 15 female and 3 male students) had come from a discipline other than nursing. For 57 (79.17%) participants, nursing was their first-choice career, for 15 (20.83%) of them it was not. Further, 22 (30.56%) participants declared there was someone in their immediate family working in healthcare, whereas 50 (69.44%) did not. After the final analysis of the text and peer-review of the concept, the themes shown in Table 2 were identified. The themes (Table 2) representing the male and female perspectives are combined for each perspective and presented separately. This was done to maintain flow and continuity in the description of the two perspectives and to emphasise the intertwining of the themes.

**Table 2:** Overview of the concept explaining gender-defined roles in nursing from two perspectives

Male nursing students’ perspective			Female nursing students’ perspective	
*Themes*	*Subthemes*		*Subthemes*	*Themes*
Reasons for choosing nursing	Altruistic	**vs.**	Altruistic	Reasons for choosing nursing
	Opportunistic		Opportunistic	
	Organisational		Organisational	
			Family and social incentives	
Gender-based perspective in academia	Nursing curriculum supports gender equality		Women-centred nursing curriculum	Gender-based perspective in academia
	Challenged gender relations		The impact of gender differences during study	
Men in nursing	Attitudes of patients		Attitudes of patients	Men in nursing
	Male nurses as team members		Male nurses as team members	
Moving beyond stereotypes	Low recognition of gender stereotyping		Efforts for gender equality in nursing	Fighting entrenched gendered cultural beliefs
	Naming ‘nurse’		Stereotypes focus more on female nurses	

### Reasons for choosing nursing

The reasons for selecting nursing as a profession may be categorised in three subthemes for the male students with an additional subtheme for the female nursing students. The first three subthemes – (a) altruistic, (b) opportunistic, and (c) organisational reasons – focus on the desire to selflessly help people, the opportunity for employment, or characteristics of the work. Another reason (d) “family and social incentives” associated with influences arising from the social environment was found for the female students.

### Male nursing students’ perspective

The male nursing students’ perspective concentrates on three themes (Table 2). The majority of the male students believed the nursing curriculum addresses the male and female perspectives on equality, with just one feeling that it is more women-centred.

Occasionally, I find that certain content reflects women’s perspective more. This is perhaps less obvious in the nursing specialities where men are more present, like emergency. (24M3F)

Most of the participating male students did not notice any differences in the approach taken by faculty staff to male or female nursing students. Two participants noted that being a minority in nursing means that male nursing students can sometimes enjoy a privileged position over their counterparts.

I have noticed that […] professors sometimes give preference to men, which is not the most common since women have always dominated this profession. I wasn’t feeling bothered by this personally yet, on the other hand, it isn’t right that this kind of gender difference is present. (23M3F)

This is not always the case, at least not during the clinical placement.

It happened that I was the only male nurse in the team during the clinical placement and I felt a little left out because the clinical mentor did not take me seriously. She always addressed us as “you girls”. I had a similar experience at one lecture. (23M3F)

Another example mentioned was the situation during clinical practice when for various reasons locker rooms were not always available separately for each gender. No participant was bothered by the fact they had to change clothes in the same room as the females, although two of them confessed having detected embarrassment and disapproval from certain female students or even female nurses working in the organisation in which the clinical practice was underway.

Male nursing students reported that men in nursing are generally well accepted by the patients, although seven of them described instances where a patient (male or female) had wanted a nurse of a specific gender, mostly female patients seeking a female nurse. This most often happened during a gynaecology and obstetrics clinical placement.

I feel that patients are more delighted to see a male nurse and they often say it is nice to see that more and more men are deciding on this profession. In most cases, the patient-student relationship is not defined by a student’s gender so much as it is by the quality of the relationship. (24M3F3)

Patients are sometimes different when we are present. They are embarrassed, especially in the gynaecology ward. On one hand, this is understandable. Their attitude to female nursing students is different. Maybe because they are women and they understand them better from this perspective. (21M3F5)

Among those who had a similar experience with female patients, there is a belief that all patients will also eventually accept men in this role, despite the current mindset. One student stated:

I have noticed that while several patients stated while washing their perineal area that this is a woman's job. I haven't heard anything similar while performing other nursing interventions. (24M3F2)

This may well explain why some male students internalise gender-defined roles and tasks in nursing at a very early stage of their career. One male student admitted:

I feel that some nursing tasks are more suited to a woman, e.g. washing the perianal area, bathing a newborn etc. (21M2F)

Men were excluded from certain interventions not only by female patients, but in some cases also by a practising nurse, even a clinical mentor.

I have noticed that they do not allow men’s presence for certain interventions in younger female patients and that this is done by female nurses. (23M3F)

Another aspect the participants mentioned based on their experience (as well as the noticeable influence of gender stereotyping) was the perceived role of male nurses as team members. Most of the participating students – male and female – felt that male nurses are more desirable team members than female nurses. A common reason given for this was the belief that male nurses are physically better suited to interventions requiring great physical strength. Other reasons were: men are more inclined to do the tasks female nurses avoid; men are seen as more discreet, understandable, helpful and skilful. Two male participants also noted that men have greater chances of professional advancement within a female nursing team.

For the large majority of male participants, the female-oriented profession does not pose any problem and, interestingly, they neither recognise nor report any direct stereotypes concerning males in nursing. One participant wrote that he had been ridiculed for the profession he has decided on.

I was bothered by the fact that I was often the target of ridicule because ‘I have chosen a female profession’. (24M3F2)

Unresolved gender issues are also embedded in language. The word nurse, in Slovenian “medicinska sestra” literally “medical sister”, is typically used as the general name for all professionals working in nursing. The name “nurse” seemed problematic for ten male students.

The name “nurse” […] I think that it was once understandable and taken for granted that this was a profession reserved for females only, however this should be changed today […]. (21M3F5)

### Female nursing students’ perspective

The analysis of female nursing students’ perspective yielded three themes (Table 2). There are some similarities with the themes explaining the male students’ perspective, together with some differences. Similar to their male colleagues, a large share of female students felt the nursing curriculum addresses gender equally, although some of them believed the curriculum is more women-centred on several levels, i.e. the content itself and the language used.

Some content is traditionally more related to the female than the male perspective, but I feel they are trying to change this. (21F3F15)

I think this is also embedded in the language. We constantly see during the lectures in PowerPoint presentations the name “nurse” referring only to female students and almost never is the male form of the word used. (21F3F5)

More than half the female students believed gender was not a barrier during their studies, yet some disagreed. For example, there were some female students who felt male nursing students were privileged by clinical mentors compared to them during the clinical placement. Two mentioned that this had also occurred at the Faculty.

(Some of the) female clinical mentors deal with male nursing students in a more privileged way than female students. I feel that they see us (females) as a threat. (22F2F4)

Female students also expressed unease when having to share a locker room to change into their work clothes. In most cases, the written response was similar “I myself didn’t have any problems with this, but I have noticed discomfort among other female students”. They also described real-life cases (arriving at clinical practice ahead of time, securing a private room for a colleague who is Muslim etc.).

More than half of the female students believe that patients treat male and female nursing students equally, although some students believe that patients would prefer to see a nurse of the same gender taking care of them. Nine students believed that some patients prefer male nurses over female ones, but often do not know what to call them.

For female nurses, you automatically hear “nurse” (in Slovenian “medicinska sestra”, in female form), but for men they (the patients) do not know what to call them. (21F3F8)

I have noticed a few comments by patients “this is a man’s job” related to the work we were doing, e.g. moving a patient’s bed or bed parts, trying to service some equipment etc. (39F3F)

Female students elaborated extensively on the role of men as team members. They based their impressions on their experiences during the clinical placement. More than half felt that men are useful as team members for their physical strength and also because they contribute to better team dynamics. Female students also emphasised other characteristics of males as team members, like a sense of responsibility, lower tendencies for conflict, and kindness as a personal characteristic. In addition, half of female students felt that having men as team members brings a ‘different’ perspective for the nursing team that is often dissimilar to the female perspective, and this is one of the reasons that 10 participants referred to while indicating they would prefer to work with a male than a female nurse.

Men in nursing are welcomed because they break the monotony of the female nursing team. They contribute with another perspective on things and are of huge help when greater physical strength is needed. They often put the patients in a better mood or motivate them […]. I think that every team should be one-half male nurses and one-half female nurses, or at least one-third of male nurses. (39F3F)

The male colleagues I had an opportunity to know were nicer, more hardworking, and more human than most of my female colleagues. (22F2F)

A few students expressed the belief that women are seen as physically inferior and thereby unable to perform certain tasks, which they stressed is not the case. Further, some also felt that women are more understandable and more ‘in touch with the situation’ than men. Female students were convinced they must resist entrenched gendered cultural beliefs in the form of stereotypes much more than male nurses must. On the other hand, the expression “nurse” (in the Slovenian language in the female grammatical form) is seen as ‘problematic’ for some of them since it puts male nurses in an unequal position.

I have only heard positive stereotypes about male nurses, but only negative ones for female nurses. (20F2F2)

I think that stereotypes in nursing are more female-focused […]. (20F2F3)

### Male vs. female nursing students’ views on their future professional roles

**Fig. 1:**
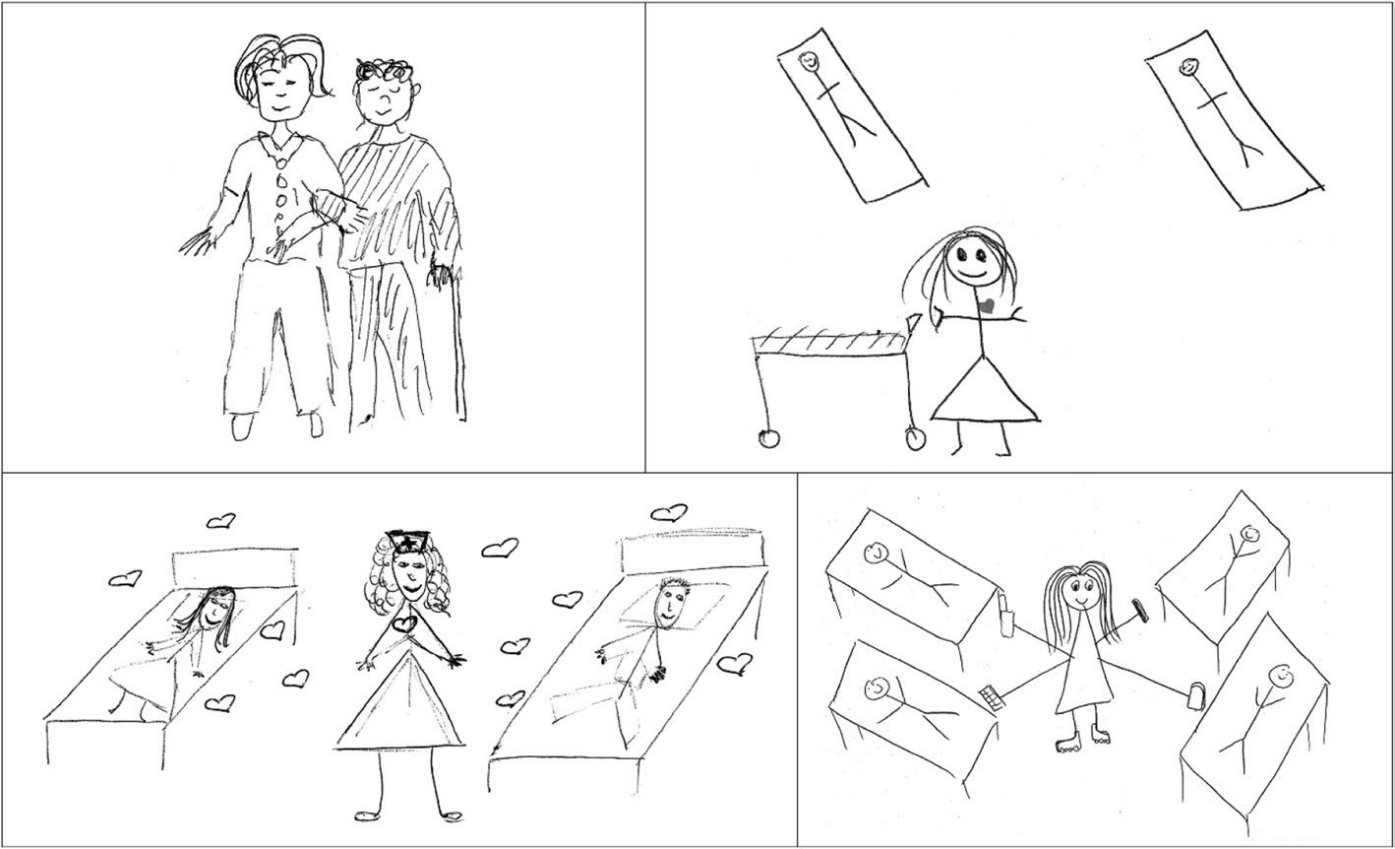
Drawing by four female nursing students

**Fig. 2:**
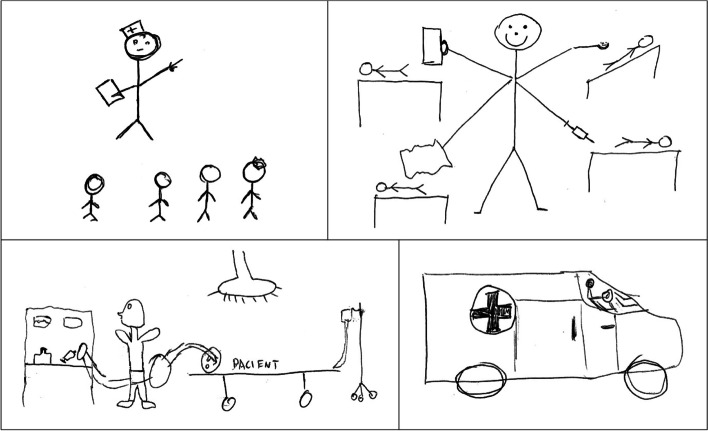
Drawings by four male nursing students

Of the 72 participating students, 61 (84.72%; 48 female and 13 male students) took the opportunity to draw how they see themselves in nursing. After coding, these drawings were arranged in four large categories. The female students’ drawings (Fig. 1) concerning their future professional role stressed a great deal of: (1) altruism; and (2) representations of a positive self-image as a nurse, while the males’ drawings (Fig. 2) showed (3) management and leadership, and the (4) technical aspects of nursing.

The females’ drawings often depicted emotional aspects of nursing through the shapes of a heart or a sun. In some cases, these two were highlighted in a black-and-white drawing with red- or yellow-coloured pencil to further stress the personal commitment to nursing and the altruistic nature of their future role as nurses. Yet, in contrast, the males’ drawings were very ‘technical’ largely featuring images of paramedics or nurse anaesthetists in their settings. While the female students depicted “multitasking”, their male colleagues’ drawings showed the delegation of tasks and similar management skills. The representation of the female and male gender (“a skirt and pants”) is clear in the drawings of either gender.

## Discussion

Our study findings show that male and female students hold a similar perspective on gender issues in nursing and are aware of various factors influencing this, yet their understanding, based on their own experience in their professional role, varies somewhat. It is obvious with the male students that, while they realise they encounter several challenges due to being a minority in a female-oriented profession, at the same time they have greater opportunities for professional advancement. Compared to the females, they are well aware of the benefits of being male in nursing and it seems they are also less burdened by stereotyping and discrimination, although this might only be because male nursing students usually pursue career choices that maintain their masculinity. Nevertheless, the findings reveal the males’ perception that they have to do more than the females, i.e. ‘to prove themselves’, not only in the profession but in the broader context as well by demonstrating certain masculine traits for which they are recognised, like strength, management, leadership and technical skills and greater knowledge [1, 4, 5]. Liu and Li [25] point out that men in nursing are becoming a minority due to the processes of gender role transformation, dual gender power struggles as well as ambiguous roles and characteristics. In this context, men must constantly redefine their identity in a female-dominated profession by gendering/de-gendering/re-gendering.

It is interesting that the male students were less burdened by the fact that female patients sometimes decline their care. Gender differences usually complicate intimate contact between nurse and patient and in our case even between the students themselves (‘the locker room case’). To reduce the associated anxiety and feelings of humiliation, male nurses must ease back on their masculinity by de-performing their gender identity [25]. Some females, but especially males, denied certain gender differences, even though other female students had confirmed their existence. According to Arreciado Marañón et al. [7], the rejection of gender difference suggests that traditional stereotypes are being deconstructed, while the affirmation of gender difference suggests their continuation.

The students who graduated from a nursing high school (secondary education) were already exposed to clinical settings’ influences compared to students who had enrolled in the bachelor’s degree nursing programme after completing other high schools, and thus had already formed certain images of gender-defined roles in nursing. In contrast, the nursing students enrolled in the bachelor’s degree nursing programme after completing other high schools described ‘idealised’ images of nursing with a less distinct definition of gender differences. These were mostly those who denied the existence of gender stereotypes in nursing or related to nursing. These findings clearly show the impact of secondary socialisation and underscore the education system’s importance for overcoming gender-defined roles in nursing [1]. Nurse educators need to identify and incorporate ways to reduce or eliminate gender barriers that students may encounter in the educational or clinical setting. Educators themselves could benefit by using non-judgmental and nonbiased strategies [6].

The prevailing patriarchal system still oppresses and limits the opportunities for male roles in nursing [1, 5, 7, 10], although the findings show that it affects females even more. The reasons for choosing nursing and the vision of one’s future professional role between the female and male students in our study are similar to those established in other studies [4, 10]. Still, more than the males, the female students were under the greater influence of their family members or social environment to select nursing as a profession, reflecting an obvious patriarchal influence. It seems that if the male role in nursing is within ‘what is acceptable’ it is thus more socially acceptable and hence less exposed to stereotyping and discrimination. Images of females in nursing are, unlike of males, much more influenced by media-generated images of nursing and nurses and many are culturally deeply-rooted in gender differences. It is therefore not only up to students to challenge these gender-based perceptions but professional associations and policymakers alike since they can seriously impact nursing shortages and recruitment, patient outcomes, the delivery of culturally-sensitive care etc. If we expect future generations of nurses to help transform nursing in the twenty-first century, we must envision nursing as a human caring task and not as an activity determined by gender. One can only hope for an ideal world of clinical placements in which there are few, if any, problems due to gender or cultural bias from patients and their families, clinical staff, students, and faculty [6].

The findings hold implications for nursing practice and nursing policy internationally. Students’ representations and perceptions of nursing are important in their choice of nursing as a profession and should be considered while planning a recruitment strategy. Given that altering gendered cultural beliefs is a long process, tackling stereotypes and raising public awareness about the role of nurses regardless of their gender would help greatly to motivate young people to decide on nursing as a career [5]. For success in these efforts, the language used both in teaching and in practice should become gender-inclusive. Namely, everyone in the profession should respectfully address nurses so that no gender is privileged and prejudices against either gender are not perpetuated.

The process of changing public opinion and thus efforts to tackle stereotypes and discrimination should initially be carried out by nurses themselves, e.g. nurses’ associations, since this forms part of the professional socialisation process and therefore has the greatest impact on candidates entering the profession. This holds further implications for the education system and indirectly for clinical settings where nursing students are exposed to various influences related to their role as a nurse. Members of faculty need to be aware, understand and mediate the gender-role stereotypes of nursing students, prevent and detect discrimination and promote gender neutrality and an integrated gender role within the curriculum [1]. Clinical settings, e.g. clinical mentors, should be particularly attentive to portraying a gender-neutral, real-life nursing role to nursing students as this impacts the transition to practice following graduation and helps prevent attrition [1, 6, 26].

The findings also suggest that appropriate strategies need to be adopted to ensure gender diversity in nursing teams and prevent gender discrimination. In clinical settings, male nurses should act more as mentors, educators, and role models to send an inspirational message to new generations choosing or entering the nursing profession [5, 6, 10]. Nursing management should also be aware of the effects of gender differences and how they clash with patients’ beliefs. A similar approach should also be adopted in academia.

### Strengths and limitations

A face-to-face interview or similar method involving more personal interaction could provide a more detailed perspective on participants’ personal experiences and allow for follow-up or supplementary questions. On the other hand, when using qualitative questionnaires, participants have the opportunity to formulate over time and to reconsider, change, and expand their answers before submitting them [19]. While the existing data collection method was also chosen because of the desire to reach a larger sample of participants using the drawing method and to increase the transferability of the findings [13], the suggested methods should be considered in future research. The researcher’s presence during the data collection, often unavoidable in qualitative research, could influence the participants’ responses, although the researcher’s absence could also permit group thinking and initiate collective consciousness and writing. Even though the study stresses the perspectives held by males and females, the researcher acknowledges that other gender identities should also be included and given attention in future research. Although studies [5, 6] show that little has changed within the nursing profession in terms of diversity, it is important to consider the impact of possible societal and professional shifts that may have influenced certain changes in gender-defined roles in nursing since the study was conducted.

The biggest concern with the drawing method is the interpretation of the findings because we rely on visual material that is more ambiguous than language-based strategies. To avoid misinterpretation or overinterpretation, we triangulated the findings with the literature and the participants’ textual narratives [27]. Despite being an understudied method, it adds great value to research by allowing the researcher to see the participants’ socially constructed reality in a more comprehensive and practical way.

## Conclusions

Nursing as a caring profession has evolved since Nightingale, but gender issues such as gender socialization in the profession and gender bias still accompany nursing. The study revealed that gender differences between male and female nursing students are not as large as one might expect, but these differences become more obvious in how their future professional roles were perceived. This has a long-term effect on the professionalisation of nursing, as male and female nurses are confined to a “gender reserved” positions within a profession. The study’s findings add to understanding of the different perspectives on gender-defined roles in nursing as well as the contextual factors that surround it. They provide a starting point for developing appropriate interventions, either in educational and clinical settings or in the public sector, by promoting nursing as a gender-neutral profession. In addition, the methodological approach used in the study provides an opportunity to further explore the ethological research tradition with the use of qualitative questionnaires and a drawing method in health care and nursing.

## Data Availability

Due to sensitive nature of the questions asked in the study, survey respondents were assured raw data would remain confidential and would not be shared publicly. The data are available from the corresponding author upon reasonable request.

## Abbreviations

RNA: Registered Nursing Assistant
EU: European Union
FHS UP: Faculty of Health Sciences University of Primorska
COREQ: Consolidated Criteria for Reporting Qualitative Research
